# Effect of green tea extract in extender of Simmental bull semen on pregnancy rate of recipients

**DOI:** 10.5713/ajas.20.0025

**Published:** 2020-04-12

**Authors:** Suherni Susilowati, Trilas Sardjito, Imam Mustofa, Oky Setio Widodo, Rochmah Kurnijasanti

**Affiliations:** 1 Department of Veterinary Reproduction, Faculty of Veterinary Medicine, Universitas Airlangga, Kampus C Mulyorejo, Surabaya-60115, Indonesia; 2 Department of Animal Husbandry, Faculty of Veterinary Medicine, Universitas Airlangga, Kampus C Mulyorejo, Surabaya-60115, Indonesia; 3 Department of Basic Veterinary Medicine, Faculty of Veterinary Medicine, Universitas Airlangga, Kampus C Mulyorejo, Surabaya-60115, Indonesia

**Keywords:** Artificial Insemination, Post-thawed Semen Quality, Progesterone, Transrectal Palpation

## Abstract

**Objective:**

The aim of this study was to ascertain the effects of adding green tea extract (GTE) to skim milk-egg yolk (SM-EY) extender on both the quality of post-thawed bull semen and the pregnancy rates of the recipient cows.

**Methods:**

Twelve ejaculates from four Simmental bulls, aged 3 to 5 years and weighing 900 to 950 kg, were diluted SM-EY extender, added with 0, 0.05, 0.1, and 0.15 mg GTE/100 mL extender and then frozen. After four weeks storage in liquid nitrogen, the sperm were thawed and evaluated for viability, motility, intact plasma membrane (IPM), and DNA fragmentation. Meanwhile, the estrus cycles of 48 recipient cows were synchronized by intramuscular administration of a single injection of 5 mg prostaglandin F2α. Estrus cows were divided into four equal groups and inseminated artificially 18 to 20 h after the onset of estrus by using semen from each extender group. Pregnancy was diagnosed by measuring serum progesterone levels at 21 days, followed by transrectal palpation 90 days after insemination.

**Results:**

The findings revealed that adding 0.1 mg of GTE/100 mL extender produced the highest percentages of sperm viability (70.67%±1.75%), motility (69.17%±1.47%), and IPM (69.23%±1.21%) and the lowest percentage of DNA fragmentation (3.00%±0.50%). The pregnancy diagnosis revealed that all cows (36/36) inseminated using frozen semen in GTE addition extender were pregnant (pregnancy rate 100%), whereas the pregnancy rate of the control group was 83.33% (10/12).

**Conclusion:**

It may be concluded that 0.1 mg GTE/100 mL extender yields the best quality of spermatozoa and that all variants doses of GTE in extender produce a higher pregnancy rate among recipient cows.

## INTRODUCTION

The problem with frozen semen is the low quality of post thawing spermatozoa, as characterized by changes in the ultrastructure and functional quality of spermatozoa, resulting in failure to fertilize the ovum [1]. This is due to the susceptibility of unsaturated fatty acids of the plasma membrane to cold temperatures during freezing. The plasma membranes of spermatozoa consist of lipids, proteins, and carbohydrates. Cryopreservation mediates changes in the fluidity of mitochondrial membranes, causing a release of reactive oxygen species (ROS), production of malondialdehyde, and changes in membrane potential; followed by protein inactivation due to ionization, lipid peroxidation, and DNA damage [2]; and, ultimately, decreased sperm fertility.

Extenders and cryoprotectants are used to prevent the disruption of spermatozoa caused by cold shock. Extenders consisting of 20% egg yolk are used widely to cryopreserve livestock sperm. Egg yolk prevents cell damage during cryopreservation mediated by the presence of high-density lipoproteins and minerals that suppress sperm cell respiration and reduce the cells’ motility. Meanwhile, the low-density lipoproteins of egg yolk prevent sperm damage by covering the sperm membrane during freezing and thawing [3]. Extenders containing a combination of skim milk and egg yolk are also used widely. Skim milk contains carbohydrates, and egg yolk contains lecithin, which can preserve the integrity of the spermatozoa lipoprotein envelope. Oxidative stress can be reduced by using plant-based antioxidants to neutralize ROS [4]. Antioxidants added to the extender can enhance spermatozoa function and reduce the harmful effects of oxidative stress caused by ROS during freezing [5]. Green tea extract (GTE) contains polyphenol, catechin, epicatechin, epigallocatechin, epicatechin gallate, and epigallocatechin-3 gallate, and it is reported to have a more potent antioxidant effect than vitamin C or vitamin E; and proven to improve the quality of post-thawed frozen semen of sheep [6]. Many studies on the effect of GTE on semen quality of bulls have been carried out [1,7]. However, the assessment of those on the Simmental bull semen and fertility test on recipient cows had never been conducted.

Therefore, this study focused on ascertaining the effect of the addition of GTE in skim milk–egg yolk extender (SM-EY) on the quality of post-thawed semen and the pregnancy rate of beef cows inseminated artificially with this semen.

## MATERIALS AND METHODS

### Animal care

The experimental procedure was approved by 520/HRECC.FODM/VII/2019 The Animal Care and Use Committee Universitas Airlangga, Surabaya, Indonesia.

### Location of study

This study was carried out at the Regional Artificial Insemination Center, a teaching farm owned by the Faculty of Veterinary Medicine, Universitas Airlangga, located at Driyo Rejo, Gresik district. The application of Simmental bull frozen semen to artificially inseminate cows was performed in the Lamongan, Situbondo, and Bondowoso Districts, all in East Java, Indonesia.

### Extraction of green tea

Green tea leaves were dried and ground to a particle size of 0.75 micrometers in a grinding machine. Green tea powder was soaked by maceration using 96% ethanol solvent, allowed to stand for three days, and covered with aluminum foil. The soaked substance was squeezed through filter paper, evaporated at 50°C in a rotary evaporator at 45 rpm to obtain a thick extract, and then freeze-dried and stored at −20°C until required [7].

### Semen collection

Four Simmental bulls, aged 3 to 5 years, and weighing 900 to 950 kg were collected using an artificial vagina, twice a week. Three ejaculates of each bull were examined both macroscopically (color, pH, volume, odor, and consistency) and microscopically (progressive individual movements, mass movements, and spermatozoa viability). If the percentage of motility and viability of spermatozoa qualify the requirements (at least 70%), the semen can be frozen. The ejaculate was processed and frozen, according to The National Standardization Agency of Indonesia [8].

### Extender preparation and freezing semen

Ten grams of skim milk powder (Merck 115338) was dissolved in 100 mL distilled water, heated to 92°C to 95°C for 10 minutes, and then cooled to 37°C. A laboratory chicken egg (CV Redjo, Surabaya, Indonesia) was cleaned, and its yolk was separated from its white. Egg yolk 5 mL was diluted in a skim milk solution to reach 100 mL in volume, and then penicillin and streptomycin were added at 1.000 IU/mL and 1 mg/mL, respectively. This SM-EY extender was divided into four equal portions for the control group (T0, without GTE), and the treatment groups T1, T2, and T3, which had 0.05, 0.1, and 0.15 mg of GTE added per 100 mL extender, respectively. Each of the extenders was divided equally into extenders A and B.

Each ejaculate of each bull was divided into four portions equally and added to extender A to obtain a concentration of 240 million spermatozoa/mL. Extender B was added glycerol to reach 16% concentration. Extender B was added slowly and gradually to extender A to obtain a concentration of 120 million spermatozoa/mL. All extended semen was equilibrated for 2 h at 5°C and then packaged in 0.25 mL semen straws at a dosage of 30 million spermatozoa/straw. The filled straws were placed on steel racks (Cooltop, Minitube, Tiefenbach, Germany), held in liquid nitrogen vapor for 10 min at −140°C, immersed immediately in liquid nitrogen (−196°C), and stored for four weeks until further assessment [7].

### Assessment of sperm quality

Twelve straws of frozen semen of each group (randomly taken one straw as representative of each ejaculate) used in this study. They were thawed in sterile water at 37°C for 30 s and divided randomly into two groups: six straws for ascertaining the sperm motility and viability, meanwhile the other six straws for plasma membrane integrity and DNA fragmentation assessment (six replications on each parameter).

#### Sperm viability

Fresh or post-thawed semen was dripped on object-glass, eosin negrosin was added and mixed homogeneously, and the mixture was smeared and dried over a flame. The semen was examined under a light microscope (Olympus BX-53, Olympus BX-53, Shinjuku City, Tokyo, Japan) at 400× magnification. The heads of live spermatozoa are transparent, whereas dead spermatozoa show damage to the plasma membrane; permeability increases gradually as the dye enters the cell, and the head appears reddish [9].

#### Sperm motility

Semen 10 μL was added with 10 μL of physiological NaCl, homogenized, and dropped on an object-glass and covered. Observation of the progressive movement of spermatozoa conducted under the light microscope at 400× magnification. Using the Computer-assisted Sperm Analyzer (CASA) method, spermatozoa motility was determined by calculating the progressive forward movement of 100 spermatozoa [9].

#### Sperm plasma membrane integrity

Hypo-osmotic solution (7.35 grams Na Citrate 2 H_2_O and 13.52 grams of fructose dissolved in 1,000 mL of distilled water) 1 mL plus 0.1 mL of spermatozoa was incubated for 30 minutes at 37°C. Observation of intact plasma membrane (IPM) was conducted under the light microscope (Olympus BX-53, Japan) at 400× magnification. Spermatozoa in which the plasma membranes are intact are characterized by circular tails, as the plasma membranes still function well at absorbing water in hypotonic environments. Spermatozoa with damaged membranes are characterized by straight tails [10].

#### Toluidine blue staining for DNA fragmentation assessment

Semen was dropped on an object-glass, smeared, dried in the air, and fixed in 96% ethanol (1:1) for 30 min at 4°C. The preparations were air-dried, hydrolyzed in 0.1 N HCl for 5 min at 4°C, rinsed three more times with distilled water, stained with 0.05% toluidine blue stain for 10 min. The preparation was washed once more with distilled water, dehydrated using t-butanol, and cleaned with xylol twice. DNA fragmentation in 100 spermatozoa was examined under the light microscope (Olympus BX-53, Japan) at 400× magnification. Bright blue spermatozoa heads indicated intact chromatin integrity (normal DNA), whereas dark blue heads indicated reduced chromatin integrity (fragmented DNA) [11].

### Estrus synchronization, artificial insemination, and pregnancy diagnosis of cow recipients

Selection of cow recipients was based on data recording and the recollection of the farmer, i.e., the cows were healthy, not pregnant, and had regular estrus cycles, body condition score 4 to 6 (9 scales), with a parity of 2 to 4. A transrectal palpation examination was performed to ascertain the presence and condition of an active corpus luteum. The estrus cycles of the qualifying cows were synchronized via intramuscular injection of 5 mg prostaglandin F2α (Enzaprost-T, Hochiminh, Vietnam). Observation of estrus was carried out two days post-injection, based on signs of nervousness or restlessness, voice, swelling, the warmth of the vulva, reddishness of vagina mucosa, thin and transparent cervical mucus expelled from the vagina, and mounting of other cows [12]. The 48 head of estrus cows were divided equally into four groups, inseminated with T0, T1, T2, and T3 post-thawed frozen Simmental bull semen. The straws of frozen semen of those groups (randomly taken one straw as representative of 12 ejaculates) thawed in sterile water at 37°C for 30 s, then used to inseminate of those 12 cows recipient of each group. The artificial insemination (AI) was conducted at 18 to 20 hours after the onset of estrus. Pregnancy was diagnosed by measuring the progesterone serum level at 21 days, followed by transrectal palpation at 90 days post AI.

### Statistical analysis

Motility, viability, IPM, and DNA fragmentation data were analyzed using analysis of variance, followed by the Tukey honestly significant difference (HSD). The results of the pregnancy diagnosis are displayed in a descriptive table.

## RESULTS

The average volume and concentration of 12 ejaculates from four Simmental bulls were 7.50±0.51 mL and 1,035±70.89 ×10^6^ spermatozoa/mL, respectively (Table 1). Mass movements were 3+, which means that spermatozoa moved together in thick, numerous, and fast waves. Each ejaculate contained 7.763±0.278×10^6^ spermatozoa, which were used to produce straws of 0.25 mL volume containing 30 million spermatozoa.

### The quality of post-thawed semen

The quality of post-thawed semen (motility, viability, IPM, and DNA fragmentation) diluted in SM-EY extender with added GTE was shown in Table 2. The addition of 0.1 mg of GTE/100 mL extender (T2) produced the highest percentages of sperm viability, motility, and IPM; in contrast, the percentage of DNA fragmentation was the lowest compared to the control group (T0), 0.05 (T1), and 0.15 (T3) mg of GTE/100 mL extender (Table 2), respectively. Post-thawed progressive motility, viability, and IPM were revealed a similar pattern: T0 (46.25%±1.53%, 48.55%±2.13%, and 43.57%± 1.21%), T1 (56.30%±1.36%, 59.00%±1.29%, and 55.67%± 1.54%), and T2 (69.17%±0.62%, 70.67%±0.71%, and 69.23% ±0.49%) were significantly different (p<0.05) among other, meanwhile T3 (52.63%±1.33%, 55.33%±0.92%, and 52.83% ±1.31%) were not significantly different (p>0.05) to T0. Post-thawed DNA fragmentation of T0 (5.33%±0.67%), T1 (4.50% ±0.43%), and T3 (4.35%±0.49%) were not significantly different (p>0.05) among other, but all of those were significantly higher (p<0.05) compared to T2 (3.00%±0.21%). This indicated that GTE 0.1 mg/100 mL in SM-EY extender resulted in the best quality of post-thawed Simmental bull sperm.

### Estrus synchronization and pregnancy diagnosis

The result of pregnancy diagnosis based on measurement of progesterone level d 21 post-AI was the same with pregnancy diagnosis through transrectal palpation (Tables 3, 4). All cows (36/36) inseminated using frozen semen in GTE addition extender were pregnant (pregnancy rate 100%), meanwhile the pregnancy rate of the control group was 83.33% (10/12).

## DISCUSSION

Addition 0.1 mg GTE/100 mL in SM-EY extender was the best quality of spermatozoa and that all variant doses of GTE in extender result in a higher pregnancy rate compared to the control group. There were two reasons for it: the excellent quality of fresh semen itself and the addition of GTE as an antioxidant. The first reason proven of the average motility of ejaculates used in the study (87.50%) was qualified according to the Indonesian National Standard [8], which states that bovine semen used for AI should have the sperm motility of fresh semen at least 70%. This Simmental bull sperm motility was better compared to the same breed bull in the earlier study was 85.83% [7], or another report was 70.2% [13]. The second reason was the addition of GTE. In this study, GTE extracted in ethanol solvent. Of different extraction solvents used, ethanol extract produced the highest yield of total catechins from green tea leaf [14] compared to the other solvent. Furthermore, the high liquid chromatography–mass spectrometry (HPLC-MS) method proved the separation and quantification of catechin in green tea leaf [15] and has shown that GTE contained catechin, epicatechin, gallocatechin, epigallocatechin, gallocatechin gallate, and epigallocatechin gallate (EGCG) [16]. EGCG was a more potent antioxidant than vitamin C or vitamin E [6]. The best of post-thawed viability, motility, and IPM were 70%, 67%, 69.17%, and 69.23% respectively; this was higher compared to an earlier report, were 68.25%, 64.35%, and 62.25%, respectively [7]. This result also better compared to an earlier study that addition of 0.75% GTE has reached motility, viability, and IPM as 52.64%, 64.32%, and 53.34%, respectively [1]. The lowest DNA fragmentation of this study (3%) was lower compared to the lowest (11.5%) observed in Bali cattle bulls [11].

The initial effect of the freezing process was on the sperm plasma membrane. The plasma membrane of spermatozoa protects organelles from mechanical damage and acts as a filter for the exchange of intracellular and extracellular substances. The integrity of the plasma membrane is vital for spermatozoa as it affects the metabolism associated with motility and viability. As motility enables spermatozoa to travel from its point of introduction to the site of fertilization, it is a significant parameter when assessing semen for AI [17]. According to the Indonesian National Standard Agency [8], frozen bovine semen used for AI should have post thawing motility of at least 40%, and DNA damage must not be greater than 5%. DNA quality plays a vital role in embryo development. Fragmentation of spermatozoa DNA due to oxidative stress can result in pregnancy failure [18].

Physiologically, spermatozoa require a low dose of ROS to play a pivotal role in events associated with sperm capacitation. However, overproduction of ROS depletes the antioxidant system in sperm, causing oxidative stress, damages sperm DNA, reducing both fertility and pregnancy rates [19]. The ROS problem can be solved by adding antioxidants, such as EGCG. The antioxidant activity of EGCG is polyphenol groups of catechin as chelating agents, whereas flavonoids could favor divesting its catalytic activity to the lipid bilayers membranes [20]. Epigallocatechin-3-gallate (EGCG) is considered the most promising bioactive compound in green tea due to its potent antioxidant activity [21]. EGCG interacts with the estrogen receptor (ER) or other proteins and/or lipids receptors in the plasma membrane, acted on signaling pathways to induced cyclic adenosine monophosphate, adenosine monophosphate-activated protein kinase, calcium ion, and ferric iron. This mechanism followed by reduced lipid peroxidation, protein carbonylation, and DNA damage; restored glutathione peroxidase and glutathione S transferase activity [20]. EGCG also affected the reduction of triglycerides content, induction of lipase, as well as the glucose-6-phosphate dehydrogenase activity, increased cholesterol efflux, and tyrosine phosphorylation. EGCG increased phosphorylation of proteins controlling cell survival such as B-cell lymphoma 2, serine/threonine-protein kinase B (Akt), and a family of non-receptor tyrosine kinases (Src) [22], Furthermore, EGCG preventing chromosomal impairment, low-density lipid oxidation, spontaneous mutation, reduced ROS level and regulation of gene expression, will further affect increased sperm motility, viability and finally improved fertility [23]. In *in vitro* experiments, either GTE or EGCG improved sperm capacitation hallmarks, including tyrosine phosphorylation and cholesterol efflux through the ER pathway [24]. This was proven in boar sperm freezing with GTE supplementation of extender, which can reduce the generation of ROS during freezing. The motility and viability of the GTE-supplemented extended semen were higher; meanwhile, the ROS level was lower compared to the control group [16].

During the thawing process, spermatozoa once again will undergo extreme changes in temperature and osmolarity that are marked by plasma membrane damage [25], including the membrane of the acrosomal cap. Acrosome disintegration and partial removal of the outer acrosomal membrane, with depletion of acrosomal content, are common alterations attributed to physical freezing events. These defects are likely due to the formation of ice crystals during the freezing of extracellular fluids, causing the sub acrosomal region to expand. Alternatively, osmotic changes may damage the structure of the lipid membrane structure, causing tension changes in water canal proteins and ionic leakage in plasma membranes, with resultant morphological changes in the cytoplasmic organs and the structure of the mitochondria [26]. Therefore, thawing at 37°C could maintain an osmose balance, improve lipid configuration, and help maintain the condition of spermatozoa plasma membrane proteins. Meanwhile, adding GTE to extender can maintain the motility, viability, intactness of the plasma membrane, and DNA integrity of the spermatozoa. In this study, the quality of post-thawed Simmental bull semen on T1, T2, and T3 still met the national standard for AI, the viability and progressive motility of spermatozoa exceeded 40%, and DNA fragmentation less than 5% [8]. That was why all of cows recipient inseminated using post-thawed semen diluted in GTE addition were pregnant. All doses of GTE in this study (T1, T2, and T3; semen diluted in SM-EY containing 0.05, 0.1, and 0.15 mg GTE/100 mL respectively) revealed a higher pregnancy rate (36/36; 100%) of recipient cows compared to control group (10/12; 83.33%) (Table 3). Higher quality of post-thawed spermatozoa is vital for attaining a higher rate of conception. GTE preserves the plasma membrane from lipid phosphorylation [7]. Intact of plasma membrane of the acrosomal cap required for the acrosomal reaction. The release of several proteolytic enzymes from the acrosome facilitates the digestion of the zona pellucida. It allows the genetic material of the sperm to enter the cytoplasm and fertilize the ovum. This conception extends the life of the corpus luteum via an interferon-τ exerting an anti-luteolytic, ensuring that progesterone levels remain high to maintain the pregnancy and useful for early pregnancy diagnosis [27].

Pregnancy diagnosis through transrectal palpation is used to confirm pregnancy from approximately day 30 of gestation [28]. The number of cows diagnosed as pregnant based on measurement of serum progesterone level was equal to the number of cows diagnosed as pregnant via transrectal palpation (Tables 3, 4). All the cows diagnosed pregnant based on progesterone levels were also diagnosed pregnant through transrectal palpation. It was indicated that there were no early embryonic deaths in cows in this study.

In conclusion, the best quality of post-thawed Simmental bull semen diluted in SM-EY extender was obtained with the addition of a 0.1 mg dose of GTE per 100 mL extender, based on high sperm viability and motility, an intact plasma membrane, and low DNA fragmentation. The rate of pregnancy among recipient cows artificially inseminated using post-thawed semen extended in a diluter containing GTE was higher than was that in the control group.

## Figures and Tables

**Table 1:** The characteristics of fresh Simmental bull semen (four bulls, three ejaculates respectively)

Parameter	Mean±SE
Volume (mL)	7.50±0.51
Consistency	Thick
Color	Yellowish white
Smell	Specific
pH	6–7
Concentration (million/mL)	1,035±70.89
Viability (%)	90.75±0.12
Mass motility	+++
Progressive individual motility (%)	87.50±0.11
Abnormalities (%)	5.25±0.14

SE, standard error.

**Table 2:** The quality of post-thawed Simmental bull semen diluted in SM-EY extender, with the addition of various doses of GTE (mean±SE) (n = 6 replication respectively1))

Parameter	T02)	T12)	T22)	T32)
Progressive (%)/mass motility	46.25±1.53^c^/2	56.30±1.36^b^/3	69.17±0.62^a^/3	52.63±1.33^c^/3
Viability (%)	48.55±2.13^c^	59.00±1.29^b^	70.67±0.71^a^	55.33±0.92^b^
Intact plasma membrane (%)	43.57±1.21^c^	55.67±1.54^b^	69.23±0.49^a^	52.83±1.31^b^
DNA fragmentation (%)	5.33±0.67^a^	4.50±0.43^a^	3.00±0.21^b^	4.35±0.49^a^

SM-EY, skim milk–egg yolk; GTE, green tea extract; SE, standard error.

1) Twelve straw of frozen semen of each group (randomly taken one straw as representative of each ejaculate) divided randomly into two groups, six straws for ascertaining the sperm motility and viability, meanwhile the other six straws for plasma membrane integrity and DNA fragmentation assessment (six replications on each parameter, respectively).

2) T0 denotes SM-EY extender without GTE, and T1, T2, and T3 denote SM-EY extender withe addition of 0.05, 0.1, and 0.15 mg GTE/100 mL extender, respectively.

a–c Different superscripts in the same row are significantly different (p<0.05).

**Table 3:** Progesterone serum level (ng/mL) of cows recipients at day 21 and the results of pregnancy diagnosis through transrectal palpation at day 90 post-AI, using frozen semen in SM-EY extender with the addition of GTE at various doses

Cow number	T01)	T11)	T21)	T31)
1	15.65/+	16.10/+	16.45/+	16.40/+
2	16.45/+	15.90/+	16.60/+	16.55/+
3	15.05/+	15.80/+	15.70/+	15.65/+
4	0.80/−	16.40/+	16.05/+	15.95/+
5	15.35/+	16.15/+	16.70/+	16.65/+
6	15.40/+	16.25/+	15.45/+	15.40/+
7	0.90/−	16.10/+	16.75/+	16.70/+
8	15.70/+	16.40/+	16.60/+	16.55/+
9	15.30/+	16.20/+	16.40/+	17.65/+
10	16.10/+	15.85/+	16.85/+	16.55/+
11	15.15/+	15.75/+	16.90/+	16.45/+
12	15.40/+	15.70/+	17.75/+	16.80/+

AI, artificial insemination; SM-EY, skim milk–egg yolk; GTE, green tea extract.

1) T0 denotes SM-EY extender without GTE; T1, T2, and T3 denote SM-EY extender with the addition of at 0.05, 0.1, and 0.15 mg GTE/100 mL extender, respectively.

+, pregnant; −, not pregnant.

**Table 4:** The average serum progesterone levels (ng/mL) at day 21 of pregnant cows (mean±SE) and the pregnancy rate of cow recipients based on transrectal palpation at day 90 post-AI using frozen semen in SM-EY extender, with the addition of GTE at various doses

Pregnancy diagnosis	T01)	T11)	T21)	T31)
Progesterone levels	15.56±0.18	16.06±0.11	16.52±0.27	16.44±0.26
Transrectal palpation (%)	83.33 (10/12)	100 (12/12)	100 (12/12)	100 (12/12)

SE, standard error; AI, artificial insemination; SM-EY, skim milk–egg yolk; GTE, green tea extract.

1) T0 denotes SM-EY extender without GTE; T1, T2, and T3 denote SM-EY extender with addition of 0.05, 0.1, and 0.15 mg GTE/100 mL extender.
